# Unraveling the Enigma of Gossypiboma: A Series of 14 Cases Highlighting the Prevalence, Root Causes, and Outcomes in Resource-Limited Settings

**DOI:** 10.7759/cureus.63856

**Published:** 2024-07-04

**Authors:** Saleh Al-Wageeh, Ismaeel A AlShoaibi, Basheer Abdo, Faisal Ahmed, Saif A Ghabisha, Mohamed Badheeb, Mohammed Ameen

**Affiliations:** 1 Department of General Surgery, Ibb University, Ibb, YEM; 2 Department of Internal Medicine, Ibb University, Ibb, YEM; 3 Department of Urology, Ibb University, Ibb, YEM; 4 Department of Internal Medicine, Yale New Haven Health, Bridgeport Hospital, Bridgeport, USA; 5 Department of Pharmacy Practice, Sana'a University, Sana'a, YEM

**Keywords:** obstetrics, labour and delivery, surgery, patient safety, adverse event, gossypiboma, retained surgical sponges

## Abstract

Background: Gossypiboma or textiloma is the unintentional retention of textile material in a patient's body, often occurring during abdominal surgery and involving surgical sponges. The body may respond to this foreign body with an exudative inflammatory reaction or an aseptic fibrotic reaction, encapsulating the cotton material and forming a mass. This rare but dramatic event can lead to life-threatening complications, and due to legal and ethical concerns, few publications exist. There were no published papers regarding this issue in our nation (Yemen). This study aims to report the retained surgical sponges' cases and their associated factors in a resource-limited setting to improve prevention.

Materials and methods: A retrospective case series study was conducted at hospitals affiliated with Ibb University, Ibb, Yemen, between March 22, 2018, and May 12, 2024. The study included 14 cases of diagnosed and surgically confirmed retained surgical sponges. Data on demographic characteristics, type of operation, and risk factors were gathered and analyzed.

Result: Among 15,120 surgical procedures, there were 14 cases of retained surgical sponges with a prevalence rate of 0.09%. The mean age was 32.5±17.0 years, with 10 (71.4%) females and 4 (28.6%) males. Gynecological surgery was the most common causal procedure (n=7, 50.0%). The most common clinical presenting features were abdominal pain in 12 (85.7%), followed by infections and a systemic reaction in 9 (64.3%). The median symptom incubation time was 37 days. 11 (78.5%) patients underwent abdominal X-rays, and 13 (92.8%) had abdominal ultrasounds, with 4 (28.6%) X-rays and 5 (35.7%) abdominal ultrasounds being deemed non-diagnostic. An abdominal-pelvic CT scan was done on 11 (78.5%) individuals, with the results being diagnostic in 10 (71.4%) and non-diagnostic in one (7.1%). The leading causes for gossypiboma occurrence were prolonged surgical procedures > one hour and emergency in 7 (50.0%) cases, followed by multiple surgical team involvement and change in nursing staff during procedures in 5 (35.7%) cases.

Conclusion: A gossypiboma or retained foreign body diagnosis can be achieved through comprehensive patient history, radiologist-surgeon interaction, understanding of risk factors, and familiarity with imaging patterns. Safety procedures should be robust and straightforward, and effective communication among surgical professionals can help minimize medical negligence and protect patients in chaotic situations. Furthermore, the surgeon should adhere to the standard prescribed method and report cases of retained surgical sponges.

## Introduction

Gossypiboma or textiloma is the unintentional retention of textile material in a patient's body, often occurring during abdominal surgery and involving surgical sponges [1,2]. This rare but dramatic event can lead to life-threatening complications, and due to legal and ethical concerns, few publications exist [3]. It has been reported to occur in 100 to 5000 of all surgical operations and one in 1000-1500 for intra-abdominal operations [4]. Furthermore, surgical sponges are responsible for the majority of foreign bodies retained. It is typically present in the abdomen (56%), pelvis (18%), and thorax (11%) [5]. The reported risk factors for gossypiboma are emergency operations, prolonged surgical procedures, multiple surgical team involvement, unexpected change in operation, simultaneously multiple major surgical procedures, incorrect instruments and gauze counts, and unexpected intraoperative difficulties with severe bleeding [2,4,6]. Gossypiboma clinical presentation is variable and may take weeks, months, or even years from the provoking surgery, with some remaining asymptomatic [5]. Radiological investigations play a principal diagnostic value, including ultrasonography (US), plain abdominal radiography, and computerized tomography (CT) scans. If the gauze has a radio-opaque marker, it can be diagnosed by plain radiography and US, but CT may provide a definitive diagnosis [6]. Gossypiboma carries a high risk of morbidity and hospital costs in the form of extended hospital stays, more investigations, and treatment [7]. Because of the legal and ethical concerns surrounding this illness, few publications are available, most of which are case reports or case series [2,8]. There were no published papers regarding this issue in our nation (Yemen). This study aims to report the retained surgical sponges' cases and their associated factors in a resource-limited setting to improve prevention.

## Materials and methods

Study design

A retrospective case series study between March 22, 2018, and May 12, 2024, conducted at hospitals affiliated with Ibb University (Althora Hospital, Al-Nasar Hospital, and Alborj Hospital), Ibb, Yemen, including 14 cases diagnosed with retained surgical sponges, which were confirmed during surgery. The study was conducted in accordance with the Declaration of Helsinki and approved by the Ibb University Institutional Ethics Committee (Code: IBBUNI. AC. YEM. 2024.78 on 2023-02-03). After thoroughly explaining the research purpose, all patients were granted informed consent to participate and release case facts and photographs.

Inclusion and exclusion criteria

All patients identified with inadvertently retained surgical sponges (gossypiboma) and confirmed surgically in general/visceral surgery, urology, and gynecological departments during the study period were included. Surgical sponges used for packing, such as vaginal packing and damage control laparotomies or those operated in other hospitals, were excluded. It should be noted that we only considered cases diagnosed at our centers; patients operating in other centers or patients received by the forensic medicine department due to a lack of information were excluded.

Collected data and definitions

The data on patients' demographic characteristics, type of operation, anatomical location, surgical specialty involved, radiologic images including (plain radiography x-ray, US, and CT scan), risk factors, and patient outcome were gathered and analyzed. Possible risk factors identified in the literature and included in this study were prolonged surgical procedures, multiple surgical team involvements, unexpected change in operation, simultaneously multiple major surgical procedures, incorrect instruments and gauze counts, unexpected intraoperative difficulties with severe bleeding, change in nursing personnel during surgery, surgery performed by trained surgeon with leadership supervision, and obesity [6]. A surgical sponge was characterized as a cotton substance (e.g., laparotomy sponge, Raytec, Cottonoid, towel, or Kerlix) introduced during an invasive surgery to absorb fluids or isolate tissue and removed before the procedure was completed [9].

Statistical analysis

The data were analyzed using IBM SPSS software (IBM SPSS, version 18, IBM Corp, Armonk, NY). Continuous variables were reported using mean and standard deviation, median, minimum (min), and maximum (mix), while categorical variables were reported as absolute numbers and percentages.

## Results

Among 15,120 surgical procedures, 14 cases of retained surgical sponges with a prevalence rate of 0.09%. The mean age was 32.5±17.0 years (3.0-67.0), with 10 (71.4%) females and 4 (28.6%) males. The characteristics of patients, clinical physical examination, radiologic findings, and the type of procedures performed are mentioned in Table 1.

**Table 1:** Characteristics of patients with retained surgical sponges, clinical, physical examination, and radiologic findings, and type of procedures performed. *Some patients had multiple symptoms.

Variables	N (%)
Age (year), mean±SD	32.5±17.0 (range: 3.0-67.0)
Gender	
Male	4 (28.6%)
Female	10 (71.4%)
Causative operation	
Hysterectomy	5 (35.7%)
Exploratory laparotomy	3 (21.4%)
Appendectomy due to perforated appendicitis	2 (14.3%)
Cesarean section	2 (14.3%)
Thyroidectomy	1 (7.1%)
Nephrectomy	1 (7.1%)
Clinical presentation*	
Abdominal pain	12 (85.7%)
Infections and systemic response	9 (64.3%)
Intestinal obstruction	5 (35.7%)
Abdominal mass	4 (28.6%)
Fistulas and discharge	2 (14.3%)
Bleeding	2 (14.3%)
Anorexia and weight loss	1 (7.1%)
Causative operation classified by the specialty	
Obstetric and gynecological surgery	7 (50.0%)
Gastrointestinal general surgery	6 (42.9%)
Urological surgery	1 (7.1%)
The time frame from operation to presentation (days), median	37 (Min: 7-Max: 360)
Radiologic image result, diagnostic	
Ultrasonography	
Diagnostic	8 (57.1%)
Not diagnostic	5 (35.7%)
Not done	1 (7.1%)
Radiography X-ray	
Diagnostic	7 (50.0%)
Not diagnostic	4 (28.6%)
Not done	3 (21.4%)
Computed tomography scan	
Diagnostic	10 (71.4%)
Not diagnostic	1 (7.1%)
Not done	3 (21.4%)
Time to second operation (days), median	42 (Min: 8-max: 361)
Specific intra-luminal site	
Vagina	4 (28.6%)
Small intestinal tract	3 (21.4%)
Stomach	2 (14.3%)
Large gastrointestinal tract	2 (14.3%)
Thorax	2 (14.3%)
Bladder	1 (7.1%)

The most common procedure was gynecological surgery (n=7, 50.0%) including hysterectomy (n=5, 35.7%) and cesarean section (n=2, 14.3%), followed by exploratory laparotomy in 3 (21.4%) cases (one pediatric case was operated on due to a foreign body swallowing (battery) in the stomach, one operated on due to a car accident with posterior urethral and bladder neck rupture, and one operated on due to gastro-vagotomy and biliary resection). Other causal surgeries were appendectomy for perforated appendicitis in two (14.3%) cases, complete thyroidectomy with neck dissection, and nephrectomy in one (7.1%) case each. Patients were presented with varied symptoms, and the most common clinical presenting features were abdominal pain in 12 (85.7%) patients, followed by infections and a systemic reaction in 9 (64.3%) patients, and intestinal obstruction in 5 (35.7%) patients. Other clinical presenting findings included abdominal mass, fistulas and discharge, bleeding, and anorexia with weight loss in four (28.6%), two (14.3%), two (14.3%), and one (7.1%) patients, respectively. The median symptom incubation time was 37 days (min: 7-max: 360 days).

**Figure 1:**
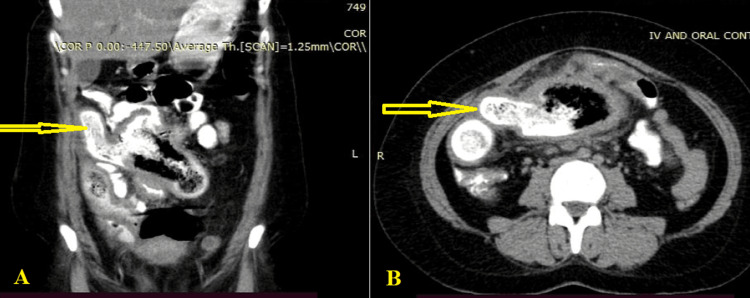
Computed tomography scan reveals a spongiform mass containing air bubbles measuring 6 × 9 × 5 cm in the intraperitoneal supraumbilical region, surrounded by a bowel loop with moderate proximal bowel dilatation suggestive of gossypiboma with partial intestinal obstruction (arrow); A: sagittal view, B: coronal view.

Regarding the radiologic study, 11 (78.5%) patients performed abdominal X-rays, 13 (92.8%) performed abdominal US, with 4 (28.6%) X-rays and 5 (35.7%) abdominal US being deemed non-diagnostic. An abdominal-pelvic CT scan was done on 11 (78.5%) individuals, with the results being diagnostic in 10 (71.4%) and non-diagnostic in one (7.1%) (Figure 1).

**Figure 2:**
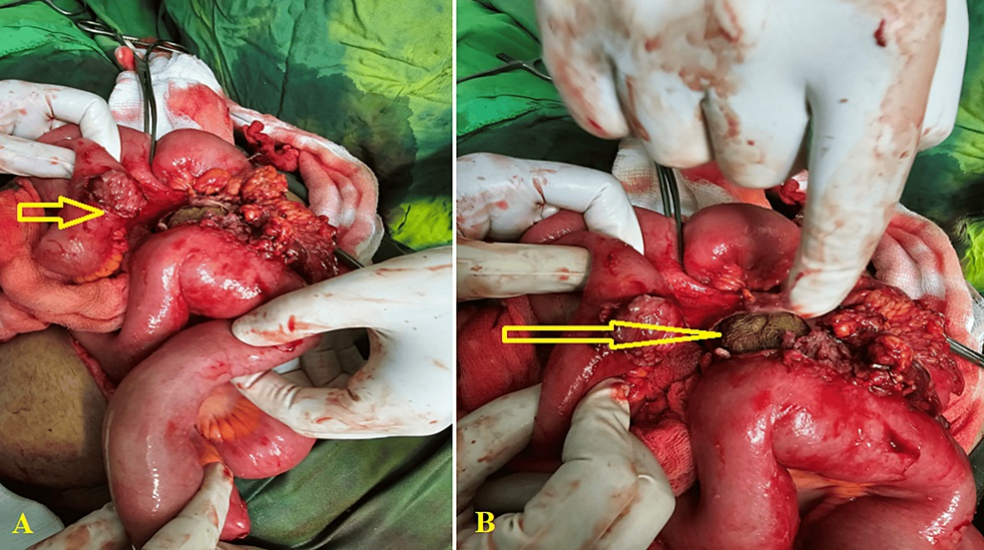
Intraoperative photos show (A) severe omental and extensive bowel adhesions, (B) a small bowel wall opening containing surgical gauze penetrates the small bowel wall and migrates to its lumen (arrow).

The median time to the second operation was 42 days (min: 8-max: 361 days). Specific intra-luminal sites of retained surgical sponges were seen in the vagina, small intestinal tract, large gastrointestinal tract, stomach, thorax, and bladder in 4 (28.6%), 3 (21.4%), 2 (14.3%), 2 (14.3%), 2 (14.3%), and 1 (7.1%) case, respectively. Adhesions occurred in 2 (14.3%) cases involving the abdominal wall, 10 (71.4%) in the intestine, and 2 (14.3%) in the colon (Figure 2).

**Figure 3:**
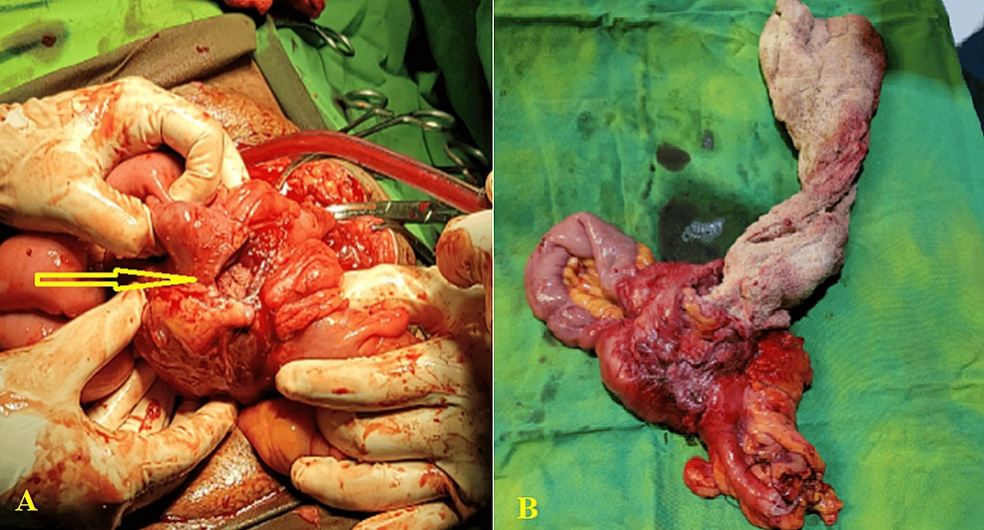
Intraoperative photos showing (A) fistula between small and large bowels contains surgical gauze (arrow). (B) En-bloc bowel resection of affected bowel segment followed by end-to-end anastomosis of booth bowel loops.

In 7 (50.0%) cases, a purulent accumulation around the sponge was observed. 2 (14.3%) cases of intestinal migration were discovered, one of which was sealed (no fistulas or peritonitis), while the other had an enteric fistula with peritonitis. Additionally, intestinal resection was performed in 2 (14.3%) cases (Figure 3).

The postoperative complication was surgical site infection in 2 (14.3%) patients, which was managed with dressing, wound irrigation, appropriate antibiotics, and reoperation in 1 (7.1%) case. There was no mortality.

Factors associated with retained surgical sponges

The main factors for retained surgical sponges were prolonged surgical procedures of more than one hour and the operation performed on an emergency basis. Both factors were reported in 7 (50.0%) cases, followed by more than one surgical team involved and change in nursing staff during procedures reported in 5 (35.7%) cases. Other factors include an unexpected change in operation, patient obesity, estimated blood loss of >500 ml or massive transfusion given before the operation, and severe bleeding >1000 ml during operations, which was reported in 3 (21.4%) for each variable. While there were no counts of sponges and instruments, performing surgery by residents without attending supervision was seen in 2 (14.3%) cases for each variable (Table 2).

**Table 2:** Risk factors for retention of retained surgical sponges. *Some patients had multiple risk factors for retention of retained surgical sponges.

Variables*	N (%)
Prolonged surgical procedure of more than one hour	
No	7 (50.0%)
Yes	7 (50.0%)
The operation was performed on an emergency basis	
No	7 (50.0%)
Yes	7 (50.0%)
Unexpected change in operation	
No	11 (78.6%)
Yes	3 (21.4%)
More than one surgical team is involved	
No	9 (64.3%)
Yes	5 (35.7%)
Change in nursing staff during procedure	
No	9 (64.3%)
Yes	5 (35.7%)
Obesity	
No	11 (78.6%)
Yes	3 (21.4%)
Estimated blood loss of >500 ml or transfusion given before the operation	
No	11 (78.6%)
Yes	3 (21.4%)
Counts of sponges and instruments	
Performed	12 (85.7%)
Performed but not sure	2 (14.3%)
Severe bleeding >1000 ml during operations	
No	11 (78.6%)
Yes	3 (21.4%)
Surgery by a trained surgeon with leadership supervision	
Yes	12 (85.7%)
No	2 (14.3%)

## Discussion

Despite recent breakthroughs in surgical procedures and technological advances targeted at patient safety in the operating room, gossypiboma, a well-known surgical complication, remains a problem in many nations [10]. It has been reported to occur in 100 to 5000 surgical operations and one in 1000-1500 intra-abdominal procedures [4]. However, this should be considered an underestimate because various factors can influence their reports, including medicolegal issues [10]. In this study, we reported the cases presented retained surgical sponges and their associated factors in a resource-limited setting. In this study, the reported prevalence of retained surgical sponges was 0.09%. In studies conducted in the early 1980s, the incidence of retained sponges and instruments was one in every 1000 and 1500 intra-abdominal procedures [11,12]. More recent research suggests an incidence of one in 5500 to one in 18,760 inpatient operations [6,13,14]. In the review by Hempel et al., the current estimate for retained surgical objects was one incident per 10,000 procedures [5]. In 49,831 general surgeries, Takahashi et al. mentioned that 24 (0.48/1000) retained foreign body events [15]. The reasons for the varied reports of retained foreign bodies include the retrospective nature of studies, a reluctance on the part of hospitals and clinicians to disclose these errors publicly due to their sensitive nature, incidental discovery of the retained foreign bodies after many years as patients may remain asymptomatic, and confidentiality requirements of insurance and legal claims hampering the publication of data on retained foreign bodies [12,16].

Gossypiboma can affect any body cavity and any surgical specialty. However, it was commonly reported in the abdominal cavity (>50%) and within obstetric and gynecological surgeries [3,17,18]. Wan et al. analyzed 254 cases of gossypiboma between 1963 and 2008. They discovered that the abdominal/pelvic cavity/vaginal vault (74%) was the most prevalent site for retained foreign bodies, followed by the thoracic cavity (11%) [19]. These findings are consistent with our report. In our study, gossypiboma commonly occurred during obstetric and gynecological surgeries. Furthermore, specific intra-luminal sites of retained surgical sponges were seen in the vagina, small intestinal tract, large gastrointestinal tract, stomach, thorax, and bladder in 4 (28.6%), 3 (21.4%), 2 (14.3%), 2 (14.3%), 2 (14.3%), and 1 (7.1%) cases, respectively.

Postoperative complications after abdominal surgeries have been associated with negative economic impact, increased morbidity, extended postoperative hospital stay, readmission, sepsis, and death [20]. The clinical presentation of gossypiboma is highly heterogeneous, and the type and severity of symptoms are attributed to the body’s reaction to retained sponge material [21]. Retained surgical sponges can cause early and late complications, including pain, infections, hemorrhage, obstruction, peritonitis, organ damage, nausea and vomiting, wound dehiscence, and delayed healing, while late complications include adhesions, fistula formation, abscess formation, bowel perforation, chronic pain, sepsis, and nutritional deficiencies [22]. The retained sponge can elicit an exudative or fibrinous reaction. As a result of local inflammation, the exudative pattern appears early in the postoperative phase. The fibrinous response develops later as the retained foreign item is encapsulated by scar tissue [8]. Abscess formation around sponge material is less common and associated with fever and increased blood inflammatory markers. Furthermore, fistula and intestinal obstruction may shortly lead to fast discovery. In rare cases, gauze may migrate downstream through the gastrointestinal tract and be expelled naturally in the feces [21]. In a comprehensive analysis of 254 case reports involving retained surgical materials, abdominal pain, and mass were the most commonly reported symptoms [5]. In this study, the most common clinical presenting features were abdominal pain (85.7%), followed by infections and systemic inflammation (64.3%), and intestinal obstruction (35.7%).

This study's median symptom incubation time was 37 days (min: 7-max: 360 days). The incubation period for symptom presentations varies and may appear within a few days or after more than 40 years of index surgery [19]. In our study, a shorter period of symptom presentation was attributed to our research being conducted in only three academic centers, with a few cases in a short follow-up period. Additionally, some cases preferred to be treated in other centers or cities, making the follow-up and recording of these cases challenging. For that, the result of our report should be interpreted with caution.

Radiopaque labeling is a technique for identifying retained foreign bodies, making sponges visible on radiographs. However, it is not generally applicable and may worsen over time. X-rays can also detect retained foreign bodies; however, they have a 10-25% false negative rate despite radiopaque markers on surgical sponges [23]. Gossypiboma detection in the US involves using an ultrasound beam to identify bright echogenic wavy structures with cystic masses. However, this procedure struggles when fat or gas is present in the abdomen [17]. Computed tomography scan is the modality of choice to exclude retained foreign bodies [12]. CT scans of surgical sponges can reveal soft-tissue tumors with bubbles, but this method can confuse gossypibomas with abscesses. These sponges are visible as soft-tissue-density masses with a whorled texture or spongiform patterns [12]. Long-term gossypiboma can cause patchy calcification and gas bubbles [24].

Furthermore, magnetic resonance imaging (MRI) and other relevant radiological techniques, such as barium contrast, may be used for the detection of the detection of retained foreign bodies. When no radio-opaque marker is seen on X-ray or CT, the unique interior structure of the gauze granuloma is best detected with MRI. It may appear as a low-signal-intensity lesion on T2-weighted imaging, with a wavy, striped, or speckled look [25]. In this study, 11 (78.5%) patients underwent abdominal X-rays, and 13 (92.8%) had abdominal US, with 4 (28.6%) X-rays and 5 (35.7%) abdominal US being deemed non-diagnostic. An abdominal-pelvic CT scan was done on 11 (78.5%) individuals, with the results being diagnostic in 10 (71.4%) and non-diagnostic in one (7.1%). The leading causes of radiology X-ray misdiagnosis are frequently due to inaccurate interpretations of metallic shadows, radiolucent materials, and unidentified foreign items. On the other hand, US misinterpretation is primarily due to unfamiliarity with textiloma imaging, its spherical shape, fluid reservoirs, and inflammatory infiltration, potentially indicating hematoma or cancer [21].

All retained surgical sponges were treated with open surgical procedures in this study. This approach was similar to previous reports emphasizing that all gossypiboma should be treated through surgical removal [21]. However, surgical intervention may not always be necessary. Spontaneous migration may occur, resulting in the ejection of foreign material through the anus during feces, the cervix, and vagina, or even the urethra [21]. Percutaneous methods for removing retained foreign bodies can be employed where they are easily accessible but inappropriate for intra-abdominal foreign bodies. There have been reports of cystoscopy and laparoscopic procedures for removing residual surgical sponges [2].

Gossypiboma is a preventable disorder that requires accurate sponge counts at the start and before the abdomen closes. Traditional manual counts are suggested, with at least two nurses counting. Swabs should be put on a stick with radiopaque markings. When in doubt, intraoperative X-rays can be employed [2]. Bar codes and electronic article surveillance systems are two new gauze tracking technologies [26,27]. The American College of Surgeons agrees with this viewpoint, emphasizing that the ideal operating room setting should allow for the focused completion of surgical tasks. A basic and effective surgical safety process, such as the WHO checklist, can also be included in institutional policies to improve surgical safety and patient outcomes. The surgical residency program curriculum and staff training should also contain basic operating room guidelines, such as proper techniques for counting surgical sponges/gauze and equipment [8]. Magnetic retrieval devices, sharp detectors, and computer-assisted detecting methods all show promise for successful metallic retained surgical sponge recovery [28]. However, they have yet to be commonly used. Cotton materials are frequently left behind despite efforts, even when accurate sponge counts are deemed correct, due to the subjective nature of the process [2].

The main risk factors associated with retained surgical sponges are complex surgical procedures, emergency surgical procedures, obesity, multiple surgical team involvement, extended surgical operations, unanticipated changes during operation, lack of resident supervision, and using small-sized sponges [18,21]. Another study found that the probability of retaining a foreign body following surgery dramatically increased in emergencies, with unanticipated changes in technique and a higher body-mass index [6]. In this study, the most common causes of retained surgical sponges were emergency surgical procedures, prolonged surgery, multiple surgical team involvement, and changes in nursing personnel during the surgery. In general, according to the 'culture of security,' the key to avoiding retained surgical sponges is widespread understanding on the part of all staff about the nature of the problem, the identification of risk factors, and the local and global assessment of the phenomena [29].

In this study, surgery performed by a trained surgeon without leadership supervision was presented as a factor of retained surgical sponges in 2 (14.3%) of cases. Our finding was similar to previous reports, such as Steelman et al., who mentioned that issues in leadership and communication were the next most frequently identified categories of contributing factors for retained surgical sponges [9]. Furthermore, Birolini et al. found that retained foreign bodies are more common in surgeons' early careers, highlighting the need for more attention for doctors in training to prevent failures [30].

Manual counting of surgical sponges is unreliable for preventing unintended retained surgical sponges, even with strict protocols. Technological systems can assist multidisciplinary surgical teams in the counting process, reducing the likelihood of unintended retained surgical sponges [31]. Studies show that barcode computerized counting systems, data-matrix systems, and radio-frequency wands can detect discrepancies and reduce unintended retained surgical sponges, with accuracy rates of 100% and 98.1%, respectively [32,33]. In this study, manual counting of all surgical sponges and other materials is usually used in our hospital, which may lead to retained surgical sponges and was presented as a factor of retained surgical sponges in 2 (14.3%) of cases. In dubious instances or surgeries without adequate sponge counts, the surgeon should extensively evaluate the surgical sites and take an abdominal X-ray before closing [34].

Study limitation

This study possesses several limitations, most notably as the relatively small sample size and retrospective design render it vulnerable to selection and misclassification biases. Furthermore, the study could not make a robust statistical analysis to determine the factors associated with retained surgical sponges. Another limitation is that we did not include other retained surgical materials in our analysis. Nonetheless, by providing our data on patients with retained surgical sponges from a resource-limited setting, our findings contribute significantly to the literature on this issue. Our result needs to be validated in a large cohort study with strict registration criteria for retained surgical sponges, including multicenter with different levels of facilities.

## Conclusions

A gossypiboma diagnosis can be achieved through a comprehensive patient history, radiologist-surgeon interaction, understanding of risk factors, and familiarity with imaging patterns. Our findings revealed that the most common causes of retained surgical sponges were emergency procedures, which are complicated by profuse bleeding and prolonged surgical procedures for more than an hour, the involvement of more than one surgical team, and a change in nursing personnel during the surgery. Safety procedures should be robust and straightforward, and effective communication among surgical professionals can help minimize medical negligence and protect patients in chaotic situations. Furthermore, the surgeon should adhere to the standard prescribed method and report cases of retained surgical sponges.

## References

[1] Biswas RS, Ganguly S, Saha ML, Saha S, Mukherjee S, Ayaz A (2012). Gossypiboma and surgeon-current medicolegal aspect-a review. Indian J Surg.

[2] Umunna J (2012). Gossypiboma and its implications. J West Afr Coll Surg.

[3] Schwappach D, Pfeiffer Y (2023). Root causes and preventability of unintentionally retained foreign objects after surgery: a national expert survey from Switzerland. Patient Saf Surg.

[4] Manzella A, Filho PB, Albuquerque E, Farias F, Kaercher J (2009). Imaging of gossypibomas: pictorial review. AJR Am J Roentgenol.

[5] Hempel S, Maggard-Gibbons M, Nguyen DK (2015). Wrong-site surgery, retained surgical items, and surgical fires: a systematic review of surgical never events. JAMA Surg.

[6] Gawande AA, Studdert DM, Orav EJ, Brennan TA, Zinner MJ (2003). Risk factors for retained instruments and sponges after surgery. N Engl J Med.

[7] George Teressa S, Saxena J, Afzal M, Morel B (2023). Surgical management of gossypiboma: a case report. Cureus.

[8] Alemu BN, Tiruneh AG (2020). Gossypiboma: a case series and literature review. Ethiop J Health Sci.

[9] Steelman VM, Shaw C, Shine L, Hardy-Fairbanks AJ (2018). Retained surgical sponges: a descriptive study of 319 occurrences and contributing factors from 2012 to 2017. Patient Saf Surg.

[10] Tchangai B, Tchaou M, Kassegne I, Simlawo K (2017). Incidence, root cause, and outcomes of unintentionally retained intraabdominal surgical sponges: a retrospective case series from two hospitals in Togo. Patient Saf Surg.

[11] Jason RS, Chisolm A, Lubetsky HW (1979). Retained surgical sponge simulating a pancreatic mass. J Natl Med Assoc.

[12] Hariharan D, Lobo DN (2013). Retained surgical sponges, needles and instruments. Ann R Coll Surg Engl.

[13] Cima RR, Kollengode A, Garnatz J, Storsveen A, Weisbrod C, Deschamps C (2008). Incidence and characteristics of potential and actual retained foreign object events in surgical patients. J Am Coll Surg.

[14] Egorova NN, Moskowitz A, Gelijns A (2008). Managing the prevention of retained surgical instruments: what is the value of counting?. Ann Surg.

[15] Takahashi K, Fukatsu T, Oki S, Iizuka Y, Otsuka Y, Sanui M, Lefor AK (2023). Characteristics of retained foreign bodies and near-miss events in the operating room: a ten-year experience at one institution. J Anesth.

[16] Lincourt AE, Harrell A, Cristiano J, Sechrist C, Kercher K, Heniford BT (2007). Retained foreign bodies after surgery. J Surg Res.

[17] Sharma G, Bigelow J (2014). Retained foreign bodies: a serious threat in the Indian operation room. Ann Med Health Sci Res.

[18] Stawicki SP, Evans DC, Cipolla J (2009). Retained surgical foreign bodies: a comprehensive review of risks and preventive strategies. Scand J Surg.

[19] Wan W, Le T, Riskin L, Macario A (2009). Improving safety in the operating room: a systematic literature review of retained surgical sponges. Curr Opin Anesthesiol.

[20] Panos G, Mulita F, Akinosoglou K (2021). Risk of surgical site infections after colorectal surgery and the most frequent pathogens isolated: a prospective single-centre observational study. Med Glas (Zenica).

[21] Modrzejewski A, Kaźmierczak KM, Kowalik K, Grochal I (2023). Surgical items retained in the abdominal cavity in diagnostic imaging tests: a series of 10 cases and literature review. Pol J Radiol.

[22] Alsuhaimi MA, Alghamdi HS, Alshaiji SA, Fayi MA, Aldhafeeri SM (2023). Retained surgical item (Gossypiboma): a case report and literature review. Ann Med Surg (Lond).

[23] Kumar GV, Ramani S, Mahajan A, Jain N, Sequeira R, Thakur M (2017). Imaging of retained surgical items: a pictorial review including new innovations. Indian J Radiol Imaging.

[24] Kopka L, Fischer U, Gross AJ, Funke M, Oestmann JW, Grabbe E (1996). CT of retained surgical sponges (textilomas): pitfalls in detection and evaluation. J Comput Assist Tomogr.

[25] Kang HS, Khoraki J, Gie J (2023). Multiphase preclinical assessment of a novel device to locate unintentionally retained surgical sharps: a proof-of-concept study. Patient Saf Surg.

[26] Greenberg CC, Diaz-Flores R, Lipsitz SR (2008). Bar-coding surgical sponges to improve safety: a randomized controlled trial. Ann Surg.

[27] Fabian CE (2005). Electronic tagging of surgical sponges to prevent their accidental retention. Surgery.

[28] Weprin S, Crocerossa F, Meyer D (2021). Risk factors and preventive strategies for unintentionally retained surgical sharps: a systematic review. Patient Saf Surg.

[29] Szymocha M, Pacan M, Anufrowicz M, Jurek T, Rorat M (2019). Leaving a foreign object in the body of a patient during abdominal surgery: still a current problem. Pol Przegl Chir.

[30] Birolini DV, Rasslan S, Utiyama EM (2016). Unintentionally retained foreign bodies after surgical procedures. Analysis of 4547 cases. Rev Col Bras Cir.

[31] Grant EK, Gattamorta KA, Foronda CL (2020). Reducing the risk of unintended retained surgical sponges: a quality improvement project. Perioperative Care Operating Room Manage.

[32] Macario A, Morris D, Morris S (2006). Initial clinical evaluation of a handheld device for detecting retained surgical gauze sponges using radiofrequency identification technology. Arch Surg.

[33] Steelman VM, Alasagheirin MH (2012). Assessment of radiofrequency device sensitivity for the detection of retained surgical sponges in patients with morbid obesity. Arch Surg.

[34] Yildirim S, Tarim A, Nursal TZ (2006). Retained surgical sponge (gossypiboma) after intraabdominal or retroperitoneal surgery: 14 cases treated at a single center. Langenbecks Arch Surg.

